# VEGF Overexpression Is a Valuable Prognostic Factor for Non-Hodgkin's Lymphoma Evidence from a Systemic Meta-Analysis

**DOI:** 10.1155/2015/786790

**Published:** 2015-02-25

**Authors:** Jing Yang, Wenlu Li, Xin He, Guofei Zhang, Lan Yue, Ying Chai

**Affiliations:** ^1^Department of Hematology, The Second Affiliated Hospital, Zhejiang University School of Medicine, Hangzhou 310009, China; ^2^College of Pharmaceutical Sciences, Zhejiang University, Hangzhou 310058, China; ^3^Department of Thoracic Surgery, The Second Affiliated Hospital, Zhejiang University School of Medicine, Hangzhou 310009, China; ^4^Department of Endocrinology, The Second Affiliated Hospital, Zhejiang University School of Medicine, Hangzhou 310009, China

## Abstract

Vascular endothelial growth factor (VEGF) plays a vital role in the progression of Non-Hodgkin's lymphoma (NHL). Although multiple studies have investigated the relationship between VEGF expression and prognosis of NHL, these studies have yielded conflicting results. Therefore, we performed a meta-analysis to evaluate the role of VEGF in the prognosis of NHL patients. We systematically searched eligible studies from databases and determined that there was a significant correlation between VEGF overexpression and overall survival (HR (hazard ratio) = 1.66, 95% CI: 1.25–2.22, *P* = 0.001). Based on subgroup analysis by study location, number of patients, the source of VEGF expression, and study design, we found that VEGF overexpression in surgically resected tissue (HR = 1.95, 95% CI: 1.41–2.69, *P* = 0.000), but not in serum (HR = 1.37, 95% CI: 0.96–1.95, *P* = 0.087), was associated with poorer prognosis. Additionally, VEGF overexpression did not correlate with performance status, LDH level, IPI score, tumor staging, B symptoms, or NHL relapse. In summary, overexpression of VEGF in lymphoma tissue represents a promising potential prognostic factor in NHL.

## 1. Introduction

Non-Hodgkin's lymphoma (NHL) is a highly heterogeneous group of lymphoproliferative malignancies arising from either lymphocytes or natural killer (NK) cells. Based on 2013 estimates from the American Cancer Society, NHL is the fifth most common human malignancy and the sixth highest cause of cancer-related deaths worldwide [1]. Currently, prognostic factors based on clinicopathological characteristics, including Ann Arbor staging and the international prognostic index (IPI), have been widely used in predicting survival of NHL patients [2, 3]. However, patients of similar tumor status and who undergo similar treatments often experience distinct prognoses. Thus, it is urgently necessary to identify individualized biological markers to more accurately predict patient outcomes so as to improve targeted therapies.

Angiogenesis is a crucial process in the growth, development, and metastasis of many tumor types, including NHL [4, 5]. Vascular endothelial growth factor (VEGF) is a prime determinant and regulator of angiogenesis, vasculogenesis, and vascular permeability [6]. VEGF family members, including VEGFA, VEGFB, VEGFC, and VEGFD, are secreted by autocrine stimulation of tumor cells as well as through paracrine influences of the proangiogenic tumour microenvironment [7, 8]. Therefore, VEGF is expected to be a useful biomarker in NHL that is associated with worse prognosis. However, the prognostic and predictive value of VEGF in NHL remains controversial due to the heterogeneity of diseases, different classifications, and methods of analysis (immunohistochemistry, enzyme-linked immunosorbent assay, etc.). Thus, it is necessary to make informed conclusions about the association between VEGF overexpression and prognosis of NHL.

In this study, we performed a meta-analysis to investigate the relationship between VEGF expression and the prognosis of NHL patients to determine whether increased VEGF expression is associated with poor clinical outcome and clinicopathologic characteristics of NHL.

## 2. Methods

### 2.1. Literature Search

We performed a systematic electronic search in PubMed and Web of Science databases using the following terms: “VEGF” and “vascular endothelial growth factor,” “Non-Hodgkin's lymphoma,” “NHL,” “prognosis,” and all possible combinations. For studies containing overlapping patients, we chose the study with the largest number of events to avoid information duplication. We also searched the references of all studies to obtain additional eligible studies.

### 2.2. Literature Selection Criteria

The inclusion criteria in the meta-analysis were as follows: (1) to provide the histologic diagnosis of NHL; (2) to include the patients untreated; (3) to investigate the relationship between VEGF expression (in serum or surgical tissue) and clinicopathological features or prognosis of NHL; (4) to measure the expression of VEGF via immunohistochemistry (IHC) or enzyme-linked immunosorbent assay (ELISA); (5) to be published in the English language. Articles published in the form of letters, case reports, reviews, and conference abstracts were excluded.

### 2.3. Data Extraction and Assessment of Study Quality

Two primary investigators (Lan Yue and Wenlu Li) independently reviewed and extracted data from eligible studies; any disagreements were resolved via further review by a third investigator (Ying Chai) until a consensus was reached. The data extracted from each study included first author, publication year, country of origin, total number of patients analyzed (VEGF positive and negative), method of VEGF detection, VEGF cut-off value, source of VEGF, treatment regimen, study design, and clinicopathological and survival data. The quality of each eligible study was assessed using the Newcastle–Ottawa quality assessment scale [9].

### 2.4. Statistical Analysis

For the pooled analysis of the relationship between VEGF expression and OS, HRs and their 95% CIs (confidence interval) were aggregated to acquire the effective value. Raw data were used if HRs and their 95% CIs were explicitly described in the article. Otherwise, Kaplan-Meier curves of OS were analyzed using Engauge Digitizer version 4.1 (http://digitizer.sourceforge.net/), and the log-rank statistic and number of events were also used to calculate HRs with 95% CIs according to the methods described by Parmar et al. [10]. ORs and their 95% CIs were combined to evaluate the relationship between VEGF expression and clinicopathological characteristics, including performance status (PS), IPI score, stage, B symptoms, LDH levels, and relapse. An observed HR > 1 indicated that patients with VEGF overexpression had worse OS, whereas an observed OR < 1 implied that patients with VEGF overexpression will be more inclined to have unfavorable clinicopathological features. The impact of positive VEGF expression on survival or clinicopathological factors was considered to be statistically significant if the 95% CI did not exceed 1.

Heterogeneity of individual HRs and ORs was assessed using the Chi-square test according to Peto's method [11]. The inconsistency index (*I* ^2^) statistic (ranging from 0% to 100%) was used to quantify the proportion of the total variation, which is due to interstudy heterogeneity rather than sampling error [12]. A *P* < 0.10 for the *Q*-test indicated the existence of heterogeneity among the studies. The pooled ORs and HRs were then calculated by the random-effects model [12]; otherwise, the fixed-effects model was adopted [13]. Begg's test was used to detect possible publication bias and a *P* value of <0.05 in Begg's test indicated the existence of publication bias. All calculations were performed using STATA version 12.0 software (Stata Corporation, Collage Station, Texas, USA) and a *P* value < 0.05 was considered statistically significant.

## 3. Results

### 3.1. Description of Studies

**Figure 1:**
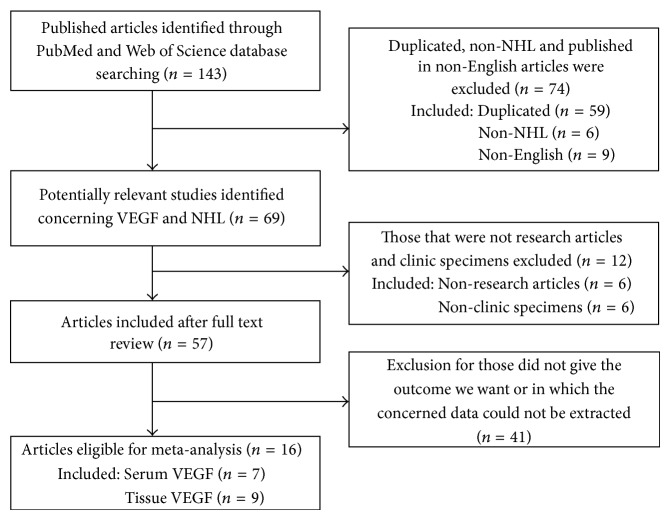
Flow diagram of studies selection procedure.

A total of 143 potentially relevant studies were retrieved by the search strategy described in Figure 1. After scrutinizing the abstracts and full text of these studies, 16 eligible studies were ultimately included in this meta-analysis [5, 14–28]. These studies were published between 2000 and 2013 and included a total of 1518 enrolled patients. Six studies were prospective and 10 were retrospective. Three studies were performed in Turkey, 3 studies in the United States, and 2 studies in Finland. Seven studies tested VEGF level in serum by ELISA, while 9 studies detected VEGF expression in surgical tissue by IHC. According to the disease subtype of NHL, DLBCL was studied in 5 studies, follicular lymphoma (FL) was evaluated in 2 studies, and peripheral T-cell lymphoma (PTL) was studied in 1 study; all others were NHL, including various subtypes such as DLBCL and FL, among others. Of the 16 eligible studies, 13 provided the HR of OS directly or indirectly. We summarized the characteristics of the 16 studies in Table 1.

### 3.2. Methodological Quality of the Studies

The quality of 16 eligible studies included in our meta-analysis was assessed according to the Newcastle-Ottawa Scale (NOS), which is widely used to evaluate the quality of case-control and cohort studies. NOS scores were calculated based on three criteria: selection, comparability, and exposure or outcome. Higher scores signified higher study quality. NOS scores of the 16 eligible studies ranged from 3 to 9. Thirteen studies received a score of greater than 5, which was indicative of a high quality study (Table 1).

### 3.3. Impact of VEGF Overexpression on OS of NHL

**Figure 2:**
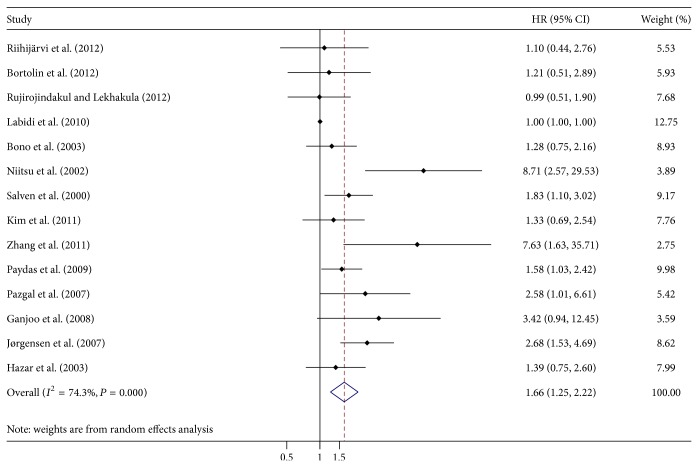
Forrest plot of Hazard ratio (HR) for the association of VEGF overexpression with overall survival (OS). HR > 1 implied worse survival for the group with VEGF overexpression.

**Figure 3:**
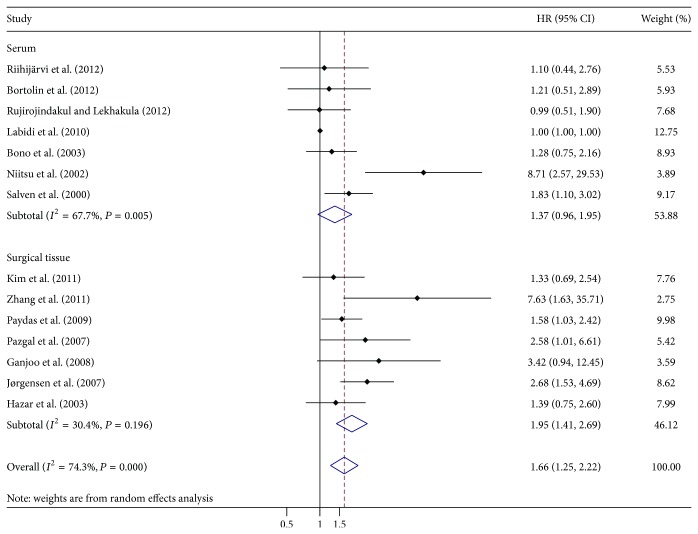
Forrest plot of Hazard ratio (HR) for the association of VEGF overexpression with overall survival (OS) according to the source of VEGF expression. Subgroup analysis showed that a significant relation between VEGF overexpression and OS was exhibited in surgical tissue.

A total of 14 studies in this meta-analysis assessed the impact of VEGF overexpression on OS of NHL. The pooled HR was 1.66 (95% CI: 1.25–2.22) (Figure 2), indicating that VEGF overexpression served as an indicator of poor OS. We also performed subgroup analysis according to study location, number of patients, source of VEGF, and study design. In the subgroup of serum-derived VEGF, the combined HR was 1.37 (95% CI: 0.96–1.95) which did not reach statistical significance (*P* = 0.087) (Table 2, Figure 3), while the combined HR of 1.95 (95% CI: 1.41–2.69) in the subgroup of surgical tissue-derived VEGF was statistically significant (*P* = 0.000) (Table 2, Figure 3). Subgroup analysis on other factors including study location, number of patients, and NOS score did not alter the significance of the prognostic impact of VEGF overexpression.

### 3.4. Correlation of VEGF Overexpression with Clinicopathological Parameters of NHL

Four studies investigated the correlation between VEGF overexpression and performance status, LDH level, and IPI score, with pooled ORs of 0.843 (95% CI: 0.39–1.882), 0.981 (95% CI: 0.636–1.51), and 0.452 (95% CI: 0.147–1.389), respectively (Table 3). The associations between VEGF overexpression and tumor staging and B symptoms were also not significant, with aggregated ORs of 0.756 (95% CI: 0.363–1.574) and 0.961 (95% CI: 0.649–1.422) (Table 3). Additionally, we also evaluated the correlation between VEGF overexpression and relapse, in which the combined OR was 0.736 (95% CI: 0.362–1.5) (Table 3). Taken together, there was no significant association between VEGF overexpression and clinicopathological features of NHL.

### 3.5. Publication Bias

**Figure 4:**
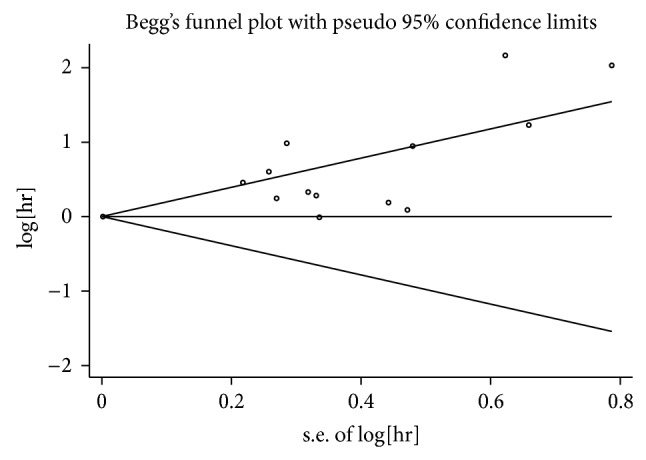
Begg's publication bias plot. The *P* value for Begg's tests is 0.477. It implies publication bias is absent for studies regarding the association of VEGF overexpression with overall survival (OS) in the meta-analysis. Each point represents a separate study for the indicated association.

The *P* value of Begg's test for VEGF overexpression on OS and clinicopathological features of NHL was 0.477, greater than 0.05, indicating the absence of publication bias in these studies (Figure 4).

## 4. Discussion

VEGF plays a crucial role in the progression of numerous tumor types, including hematopoietic malignancies [29–31]. First, VEGF can stimulate angiogenesis and lymphangiogenesis and increase vascular permeability, which is associated with reduced drug delivery and tumor cell metastasis [14, 32]. Second, VEGF induces activation of antiapoptotic genes, including bcl-2, which protect tumor cells from apoptosis [33]. Third, VEGF works in concert with numerous signaling molecules such as angiopoietins, ephrins, hepatocyte growth factor, hypoxia-inducible factor, IL-6, and endostatin to promote tumor cell survival [34–38]. Third, VEGF impacts hematopoiesis by blocking the differentiation of multiple hematopoietic lineages and inhibits the maturation of dendritic cells by reducing NF-*κ*B activation [32, 39, 40]. It has been also reported that VEGF overexpression is an indicator of poor prognosis in breast carcinoma, lung cancer, and hematopoietic malignancies [41–43]; however, the correlation between VEGF expression in NHL and patient prognosis remains unclear. Thus, we performed a quantitative meta-analysis to determine the association between VEGF expression and the prognosis of NHL.

Our meta-analysis revealed that VEGF overexpression was significantly associated with poorer prognosis of NHL (HR = 1.66, 95% CI: 1.25–2.22, *P* = 0.000), but not with clinicopathological features of NHL, such as performance status (OR = 0.843, 95% CI: 0.39–1.882, *P* = 0.640), LDH level (OR = 0.981, 95% CI: 0.636–1.51, *P* = 0.930), IPI score (OR = 0.452, 95% CI: 0.147–1.389, *P* = 0.170), tumor staging (OR = 0.756, 95% CI: 0.363–1.574, *P* = 0.450), B symptoms (OR = 0.961, 95% CI: 0.649–1.422, *P* = 0.840), or relapse (OR = 0.736, 95% CI: 0.362–1.5, *P* = 0.400). The results of our study were in accordance with those of scholars Zhang et al., which even identified VEGF overexpression as an independent prognostic factor through multivariate survival analysis [22]. These results provide rationale to support efforts targeting VEGF in NHL. Bevacizumab is the most effective monoclonal antibody to therapeutically target VEGF [44]. In a phase II trial, 11 of 45 patients with relapsed and aggressive NHL exhibited prolonged stable disease and median time of response after a single treatment with bevacizumab [45]. Furthermore, bevacizumab combined with conventional chemotherapeutics was also shown to be safe and effective in newly diagnosed diffuse large B cell lymphomas [46]. Subgroup analysis further showed that VEGF overexpression in surgical tissue (HR = 1.95, 95% CI: 1.41–2.69, *P* = 0.000) but not in serum (HR = 1.37, 95% CI: 0.96–1.95, *P* = 0.087) negatively correlated with OS of NHL. It is likely that the VEGF derived from lymphoma tissue only accounts for a minor extent in the serum [16]. In addition, serum VEGF levels in patients with different extents of disease are highly variable, making it difficult to obtain a single cut-off value as a predictor in all NHL patients [14]. Additional clinical trials are warranted to further verify the relationship between the serum VEGF levels in patients with varying degrees of disease in NHL.

We acknowledge several limitations exist in our study. Firstly, there was significant heterogeneity among the 16 studies included in this meta-analysis. Although the random-effects model was used to reduce the influence of heterogeneity, the model did not identify the source of heterogeneity. In order to clarify the source of heterogeneity, differences in study location, number of patients, source of VEGF, and NOS score were analyzed. When the analysis of OS was performed without consideration of these factors, heterogeneity was detected (*I* ^2^ 74.3% *P* = 0.000); however, when the analysis was limited to studies of surgical tissue, no heterogeneity was found (*I* ^2^ 30.4% *P* = 0.196). When the analysis of OS was limited to studies of serum VEGF levels, heterogeneity still existed (*I* ^2^ 66.7% *P* = 0.005), suggesting that the source of VEGF contributes to heterogeneity in our results. In addition, selection bias may be caused by exclusion of non-English articles. Furthermore, although various methods had been used to acquire the primary data we needed, the OS of three studies was still absent; it would inevitably result in evaluability bias. Additionally, univariate prognostic value was included in our analysis due to the limited data provided. Finally, the reliability of the results of prospective cohort studies or retrospective case-control studies selected in our study is lower than that of prospective randomized trials.

In conclusion, this meta-analysis is the first to explore the correlation between VEGF overexpression and survival and clinicopathological features of patients with NHL. VEGF overexpression in surgical tissue rather than in serum significantly correlated with worse overall survival in NHL whereas there was no relationship between VEGF overexpression and clinicopathological characteristics of NHL. Consequently, identify the expression of VEGF in surgical tissue instead of serum may be beneficial for patients before their target therapy. Further adequately designed prospective studies, however, are still necessary to strengthen the results presented here.

## Figures and Tables

**Table 1:** Characteristics of studies included for the meta-analysis.

First author	Year	Country	Patient (P/N)	VEGF source	Method	Disease type	Method to determine the threshold	VEGF positive threshold	HR (95% CI) of OS	Therapy regimen	Quality score	Study type
Riihijärvi [16]	2012	Finland	102 (25/77)	Serum	ELISA	DLBCL	Highest quartile	947 pg/mL	1.096 (0.435–2.762)	R-CHOEP + methotrexate, cytarabine	9	P

Bortolin [15]	2012	Italy	68 (33/35)	Serum	ELISA	NHL	Median value	250 pg/mL	1.21 (0.51–2.89)	ACVBP/CHOP/CHOP like + partial rituximab	6	P

Rujirojindakul [14]	2012	Thailand	79 (NA)	Serum	ELISA	NHL	Median value	516 pg/mL	0.99 (0.51–1.9)	CHOP/R-CHOP	7	P

Labidi [17]	2010	France	60 (NA)	Serum	ELISA	FL	Median value	138 pg/mL	1.002 (0.999–1.005)	CHOP like	5	R

Bono [18]	2003	Finland	143 (46/97)	Serum	ELISA	NHL	Highest tertile	465 pg/mL	1.28 (0.75–2.16)	CHOP/CHOP like	6	P

Niitsu [19]	2002	Japan	149 (73/76)	Serum	ELISA	NHL	Median value	242 pg/mL	8.71 (2.57–29.53)	CHOP/CHOP like	8	P

Salven [20]	2000	America	200 (50/150)	Serum	ELISA	NHL	Highest quartile	462 pg/mL	1.83 (1.1–3.02)	Bleo-CHOP/M-BACOD	6	R

Kim [21]	2011	Korea	51 (30/21)	Surgical tissue	IHC	DLBCL	Percentage of positive cells	Any staining	1.329 (0.695–2.541)	Methotrexate-based chemotherapy + WBRT	6	R

Zhang [22]	2011	China	38 (31/7)	Surgical tissue	IHC	PTL	Intensity score and percentage of positive cells score	Multiplying the intensity core by expressions core >2	7.633 (1.634–35.714)	CHOP/CHOP like	9	P

Paydas [23]	2009	Turkey	177 (108/69)	Surgical tissue	IHC	NHL	Percentage of positive cells	10%	1.578 (1.03–2.418)	Anthracycline containing regimens	7	R

Citak [24]	2008	Turkey	25 (10/15)	Surgical tissue	IHC	NHL	Percentage of positive cells	Any staining	NA	BFM, LMT regimens	7	R

Gratzinger [25]	2008	America	172 (75/97)	Surgical tissue	IHC	DLBCL	Percentage of positive cells	30%	NA	CHOP/CHOP like	8	R

Pazgal [26]	2007	Israel	36 (21/15)	Surgical tissue	IHC	DLBCL	Percentage of positive cells	30%	2.579 (1.007–6.606)	CHOP/CHOP like	6	R

Ganjoo [27]	2008	America	44 (22/22)	Surgical tissue	IHC	DLBCL	Percentage of positive cells	10%	3.421 (0.941–12.447)	CHOP	5	R

Jørgensen [5]	2007	Denmark	103 (64/49)	Surgical tissue	IHC	FL	Staining pattern	Diffuse pattern	2.682 (1.533–4.693)	CHOP like + radiotherapy	7	R

Hazar [28]	2003	Turkey	71 (24/47)	Surgical tissue	IHC	NHL	Percentage of positive cells	Any staining	1.391 (0.745–2.597)	Na	4	R

P/N, the number of positive/negative VEGF expression; VEGF, vascular endothelial growth factor; IHC, immunohistochemistry; ELISA, enzyme linked immunosorbent assay; DLBCL, diffuse large B-cell lymphoma; NHL, Non-Hodgkin lymphoma; FL, follicular lymphoma; NA, not available; HR, hazard ratio; 95% CI, 95% confidence interval; OS, overall survival; P, prospective; R, retrospective.

R-CHOEP, rituximab, cyclophosphamide, doxorubicin, vincristine, etoposide, and prednisone; ACVBP, doxorubicin, cyclophosphamide, vindesine, bleomycin, and prednisone; CHOP, cyclophosphamide, doxorubicin, vincristine, and prednisone; M-BACOD, methotrexate, bleomycin, doxorubicin, cyclophosphamide, vincristine, and dexamethasone; WBRT, whole brain radiation therapy.

**Table 2:** Stratified analysis of pooled hazard ratios of NHL patients with VEGF overexpression.

Stratified analysis	Number of studies	Number of patients	Pooled HR (95% CI)	*P* value	Heterogeneity		Interaction *P* value
					*I* ^2^ (%)	*P* value	
Study location							0.512
Asia	7	798	1.9 (1.21–2.97)	0.005	59.6	0.021	
Europe and America	7	720	1.5 (1.03–2.18)	0.034	72.6	0.001	
Number of patients							0.311
>100	6	874	1.88 (1.29–2.75)	0.001	56.3	0.043	
<100	8	644	1.42 (1-2)	0.047	56.2	0.025	
Source of VEGF							0.165
Serum	7	801	1.37 (0.96–1.95)	0.087	67.7	0.005	
Surgical tissue	7	717	1.95 (1.41–2.69)	0	30.4	0.196	
NOS score							0.288
>5	11	1343	1.75 (1.3–2.36)	0	49.3	0.032	
≦5	3	175	1.27 (0.78–2.06)	0.333	55.9	0.104	

**Table 3:** VEGF overexpression and clinicopathological features of NHL.

Clinicopathological features	Number of studies	Number of patients	Analytical model	Pooled OR (95% CI)	*P* value	Heterogeneity	
						*I* ^2^ (%)	*P* value
Performance status (ECOG) (0-1 versus *⩾* 2)	4	502	REM	0.84 (0.39–1.88)	0.64	67.8	0.025
LDH level (normal versus elevated)	4	502	FEM	0.98 (0.64–1.51)	0.93	5	0.37
IPI score (0–2 versus 3–5)	4	383	REM	0.45 (0.15–1.39)	0.17	78.3	0.003
Tumor staging (I II versus III IV)	5	483	REM	0.76 (0.36–1.57)	0.45	62.4	0.03
B symptom (absent versus present)	5	547	FEM	0.96 (0.65–1.42)	0.84	0	0.61
Relapse (no versus yes)	3	253	FEM	0.74 (0.36–1.5)	0.4	0	0.72
